# Lysis Physiology of *Pseudomonas aeruginosa* Infected with ssRNA Phage PRR1

**DOI:** 10.3390/v16040645

**Published:** 2024-04-21

**Authors:** Rimantas Daugelavičius, Greta Daujotaitė, Dennis H. Bamford

**Affiliations:** 1 Department of Biochemistry, Vytautas Magnus University, LT-44248 Kaunas, Lithuania; greta.daujotaite@thermofisher.com; 2 Molecular and Integrative Biosciences Research Programme, Department of Biological and Environmental Sciences and Institute of Biotechnology, University of Helsinki, FIN-00014 Helsinki, Finland

**Keywords:** bacteriophage PRR1, *P. aeruginosa*, electrochemical monitoring, ATP level, aeration

## Abstract

The phage PRR1 belongs to the *Leviviridae* family, a group of ssRNA bacteriophages that infect Gram-negative bacteria. The variety of host cells is determined by the specificity of PRR1 to a pilus encoded by a broad host range of IncP-type plasmids that confer multiple types of antibiotic resistance to the host. Using *P. aeruginosa* strain PAO1 as a host, we analyzed the PRR1 infection cycle, focusing on cell lysis. PRR1 infection renders *P. aeruginosa* cells sensitive to lysozyme approximately 20 min before the start of a drop in suspension turbidity. At the same time, infected cells start to accumulate lipophilic anions. The on-line monitoring of the entire infection cycle showed that single-gene-mediated lysis strongly depends on the host cells’ physiological state. The blockage of respiration or a reduction in the intracellular ATP concentration during the infection resulted in the inhibition of lysis. The same effect was observed when the synthesis of PRR1 lysis protein was induced in an *E. coli* expression system. In addition, lysis was strongly dependent on the level of aeration. Dissolved oxygen concentrations sufficient to support cell growth did not ensure efficient lysis, and a coupling between cell lysis initiation and aeration level was observed. However, the duration of the drop in suspension turbidity did not depend on the level of aeration.

## 1. Introduction

The phage PRR1 belongs to the *Leviviridae* family, a group of ssRNA bacteriophages that infect Gram-negative bacteria. PRR1 is notable in the group for its unique ability to infect a wide variety of host cells, such as *Pseudomonas aeruginosa*, *Pseudomonas fluorescens*, *Pseudomonas putida*, *Escherichia coli, Salmonella enterica*, and others. Susceptibility is determined by the specificity of PRR1 to a pilus encoded by a broad host range of IncP-type plasmids that confer multiple types of antibiotic resistance to the host [1].

The bacterial envelope must be permeabilized to release the reproduced phage particles. Filamentous bacteriophages, such as M13, f1, Pf3, B5, and others, use a sophisticated secretion apparatus and, because of their unique morphology and morphogenesis, are continuously extruded from their host without any lethal effect on the cell [2,3]. However, all other bacteriophages must either degrade or otherwise compromise the barrier of the bacterial envelope to accomplish their vegetative cycle [4,5]. At least two different strategies exist for phage-caused host lysis. The infection cycle of most bacteriophages containing a double-stranded DNA genome and infecting Gram-negative bacteria is finalized by the holin–endolysin–spanin system [2,4,5]. Endolysin is a generic name for a soluble phage-encoded muralytic enzyme. Usually, endolysins lack secretory signals and accumulate in the cytosol, where they are unable to access their substrate, the peptidoglycan layer. Some endolysins are exported outside the plasma membrane (PM) by the host’s *sec* system but remain relatively inactive until the PM is energized [2,5]. To degrade the cell’s peptidoglycan layer, endolysins require a second factor, a small membrane protein called holin, which forms PM lesions. These lesions either permeabilize the PM, allowing the endolysins access to the peptidoglycan, or activate the pre-exported endolysins, depolarizing the PM [2,4,5].

In contrast, endolysins are not encoded or produced by three classes of very small and simple lytic phages with single-stranded genomes: the ssDNA *Microviridae* (~5.4 kb genome), the ssRNA male-specific *Leviviridae* (~3.5 kb), and *Alloleviridae* (~4.2 kb), which are represented by the phages φ ϕX174, MS2, and Qβ [6,7,8]. In these cases, a single phage gene—*E* for ϕX174, *L* for MS2, and *A*_2_ for Qβ—was shown to be necessary and sufficient for host cell lysis. However, none of these gene products are associated with active cell wall degradation. Genetic and biochemical evidence was provided to establish the molecular basis for the lytic function of the ϕX174 E protein [9,10] and the A_2_ protein of Qβ [2,8]. In both cases, the lysis proteins were shown to inhibit a specific enzyme in peptidoglycan synthesis. The mechanism of the protein L-mediated lysis of phage MS2-infected cells remains unknown, but recent studies have shown that the interaction of this protein with the cell envelope induces lesion formation in the outer membrane (OM), followed by a disruption of the peptidoglycan layer and the disintegration of the PM [11,12]. 

From previous studies with penicillin-induced cell lysis, it is clear that arrest in peptidoglycan synthesis disturbs the well-orchestrated action of enzymes participating in the assembly of the cell wall [13]. Several of these enzymes, called autolysins, have peptidoglycan-degrading activity. These enzymes must be tightly regulated to prevent uncontrolled cell wall degradation, commonly called autolysis [13]. The inhibition of peptidoglycan synthesis triggers the uncontrolled activity of autolysins, proposing a possible mechanism of single-gene-mediated cell lysis [2,4]. However, although it is under the same regulatory control mechanisms as penicillin-induced lysis, L protein is not involved in the inhibition of peptidoglycan synthesis [14].

More than 15 years ago, the complete genome sequence of PRR1 (3573 nt) was reported [15]. Four genes, encoding maturation, coat, replicase, and lysis proteins, were identified. The genome organization and amino acid sequences of all of the proteins are similar to those of the members of the genus *Levivirus*, except for the coat protein, which resembles those of the members of *Alloleviridae*. As in the other known lysis proteins of single-gene-mediated lysis, the PRR1 lysis protein has one predicted transmembrane helix at the C-terminus [15]. The activity of the lysis gene was confirmed by the insertion of the corresponding nt region (nt 1780–1944) into an expression vector. Cell lysis was observed upon induction, indicating that the expression of the lysis gene is sufficient for cell disruption.

In addition to the analysis of global gene expression changes caused by PRR1 infection [16], we examined the PRR1 infection cycle, using *P. aeruginosa* as a host and focusing in particular on cell lysis. *Pseudomonas infections* can develop at many anatomic sites, including the skin, urinary tract, lungs, bones, ears, eyes, and heart valves. *P. aeruginosa* infections are becoming more difficult to treat because of increasing antibiotic resistance, and phage therapy looks like a promising alternative. The efficiency of the assembly of infective viral particles as well as physiological changes in the host were monitored during one-step growth experiments. In addition, we demonstrated that the efficiency of lysis is strongly dependent on the aeration of the infected *Pseudomonas aeruginosa* culture. The same effect was observed when the synthesis of the PRR1 lysis protein was induced in an *E. coli* expression system.

## 2. Materials and Methods

Bacterial strains and PRR1 propagation. *Pseudomonas aeruginosa* strain PAO1 [17] was cultivated in Luria-Bertani broth (LB, Thermo Fisher, Waltham, MA, USA) and used as a host for PRR1 propagation [1]. Either PRR1 was used as a fresh stock or the virus was purified as described below. Stocks were prepared by adding LB (3 mL/plate) to soft agar collected from semi-confluent plates and incubating for 6 h at 37 °C with aeration; debris was removed by low-speed centrifugation (10,000 rpm, 20 min, Sorvall SS-34 rotor, 4 °C). The stock titer was determined by plating dilutions on lawns of PAO1 cells. When growing cells in flasks, 1/10 of the flask volume was filled with LB medium. 

The PRR1 lysis gene was inserted into the plasmid pJJ2 [15] to produce the plasmid pTR117, which was transferred to *Escherichia coli* HMS174(DE3). For the cultivation of HMS174(DE3)(pJJ2) and HMS174(DE3)(pTR117) cells, LB was supplemented with ampicillin (150 μg/mL).

PRR1 concentration and purification. For the production of PRR1 particles, PAO1 cells were grown at 37 °C to ~7 × 10^8^ CFU/mL and infected with a fresh agar stock using a multiplicity of infection (MOI) of 20. The culture was incubated at 37 °C for 3–4 h until complete lysis occurred. DNase I (Promega, Madison, WI, USA) was added to a final concentration of 85 μg/mL, and the lysate was further incubated at 37 °C for 45 min. The lysates were cleared by low-speed centrifugation (7000 rpm, 30 min, Sorvall SLA3000 rotor, 4 °C). Virus particles were precipitated by adding solid PEG 6000 (Sigma-Aldrich, St. Louis, MO, USA, 10% final concentration) and NaCl (0.5 M final concentration), followed by magnetic stirring for 30 min at 4 °C. Phage precipitates were collected by low-speed centrifugation (7000 rpm, 20 min, Sorvall SLA3000 rotor, 4 °C), and the pellets were allowed to dissolve overnight in 20 mM of potassium phosphate buffer at pH 7.4 (approximately 3 mL/L of lysate) and 4 °C. Phage particles were purified by rate zonal centrifugation in a 5–20% (*w*/*v*) sucrose gradient (20 mM of potassium phosphate buffer, pH 7.4; 24,000 rpm, 3 h, Sorvall AH629 rotor, 15 °C). The gradient resulted in light-scattering virus zones, from which virions were recovered by differential centrifugation (32,000 rpm, 17 h, Sorvall T647.5 rotor, 4 °C). Pellets were resuspended in 20 mM of potassium phosphate buffer (pH 7.4; approximately 0.3 mL/L lysate) on ice for at least 6 h. The number of infective phage particles was determined by plating the samples on lawns of PAO1 cells using the soft agar method. The soft agar contained 7 g of agar per liter of LB broth, whereas the nutrient agar contained 15 g of agar per liter of LB broth.

Lysis profile determination experiment. For one-step growth experiments, PAO1 cells were grown overnight at 37 °C on LB plates containing 10.8 g of agar per liter of LB broth. A single colony was resuspended in 5 mL of LB, diluted ~40 fold to an A_550_ of 0.1, and grown on a shaker (200 rpm/min) in 100 mL of LB medium containing 1 l flasks to an A_550_ of 1 (corresponding to about 7 × 10^8^ CFU/mL). Phage was added to obtain a desired MOI, and the culture was further incubated under the same conditions until complete lysis occurred. Cell growth and lysis were ascertained by measuring culture turbidity (A_550_) using a Selecta Clormic digital spectrophotometer (J.P.Selecta). To determine virus production, bacterial debris was removed by centrifugation (13,000 rpm, 5 min, Heraeus Biofuge, at room temperature), and the number of plaque-forming units (PFUs) in the supernatant was determined.

Artificial lysis and determination of intracellular number of phage particles. The ability of chloroform or lysozyme to cause lysis was examined by adding chloroform (Sigma-Aldrich, 1–2% *v*/*v*), lysozyme (Merck, final concentration 50 μg/mL), or both to infected or uninfected cultures. The subsequent drop in turbidity was monitored until a constant value was obtained. In the case of infected cells, samples were withdrawn for centrifugation in a Heraeus Biofuge (13,000 rpm, 5 min at room temperature), followed by measuring the number of infective particles.

Solid-phase, overnight-grown PAO1 cells were resuspended, diluted to an A_550_ of 0.1, and further grown at 37 °C with aeration until ~7 × 10^8^ CFU/mL (A_550_ 1.0) was reached. The culture was infected (MOI 20) and incubated under the same conditions for 15 min to ensure that every cell was infected. The culture was vigorously shaken to detach the pili and remaining viruses from the cell surface. Then, the unadsorbed phage particles were separated from the cells by centrifugation (4000 rpm, 20 min, Eppendorf A-4-62 rotor, 37 °C). The cell pellets were gently resuspended in the initial volume of LB preheated to 37 °C. Infectious centers were determined, and the cell suspension was divided into aliquots and further incubated at 37 °C with aeration. At different time points, artificial lysis was initiated. Cell debris was removed (13,000 rpm, 5 min, Heraeus Biofuge, room temperature), and the number of progeny viruses was determined by plating the samples on the lawns of PAO1 cells using the soft agar method.

Thin-section electron microscopy. Bacterial cells were grown, infected, collected by centrifugation, and resuspended, as described for the determination of the intracellular number of phage particles. Samples for EM were taken at 60, 100, and 160 min post-infection (p.i.). Then, 3 mL of culture was mixed with 3 mL of 6% glutaraldehyde. After fixation for 20 min at room temperature, cells were collected by centrifugation (4000 rpm, 15 min, Eppendorf A-4-62 rotor, 28 °C), washed with 1 mL of 20 mM potassium phosphate buffer (pH 7.2), and pelleted (13,000 rpm, 5 min, Heraeus Biofuge, room temperature). Samples were prepared for transmission electron microscopy as previously described [18]. The micrographs were taken with a JOEL 1200 EX electron microscope (at the EM unit, Institute of Biotechnology, University of Helsinki) operating at 60 kV. 

Electrochemical measurements. The PAO1 cells used for electrochemical studies were grown overnight on LB plates at 37 °C, resuspended in LB medium to obtain an A_550_ of 0.1, and grown to an A_550_ of 0.8. Then, 30 mL of cell aliquots were transferred into thermostated (37 °C) and magnetically aerated 50 mL reaction vessels, grown to an A_550_ of 1, and infected with purified PRR1 at MOI 20. For dissolved oxygen measurements, PAO1 cells were grown as usual, collected by centrifugation (8000 rpm, 20 min, Sorval SLA1500 rotor, 4 °C), and resuspended in LB to ~7 × 10^10^ CFU/mL. 

In the case of *E. coli* HMS174(DE3) cells containing a recombinant plasmid, the overnight culture was grown with aeration at 37 °C, diluted to an A_550_ of 0.1, and further grown to an A_550_ of 0.7. Then, gene expression was induced by adding isopropyl-β-D-thiogalactopyranoside (IPTG, Merck, Rahway, NJ, USA) to a final concentration of 1 mM.

The concentrations of phenyldicarbaundecaborane (PCB^−^) and K^+^ ions and the amount of dissolved oxygen in the medium were monitored by selective electrodes. The K^+^-selective electrode (Orion model 93-19) and the dissolved oxygen electrode (Orion model 970800) were from Thermo Inc. The construction and characteristics of the PCB^-^-selective electrode have been described previously [19,20]. For K^+^ and PCB^−^ measurements, Ag/AgCl reference electrodes (Thermo Inc.; Orion model 9001) were indirectly connected to the measuring vessels through an agar salt bridge. 

The measurements were performed simultaneously in 3 or 4 reaction vessels. For PCB^-^ measurements, 30 nmol of PCB^-^ was added to 30 mL of the culture before infection. The electrodes were calibrated at the end of every experiment. When oxygen was measured, solid Na_2_S_2_O_5_ was added at the end of each experiment to exhaust the dissolved oxygen. The oxygen concentration in the medium before the cell addition is referred to as 100%, while the concentration after Na_2_S_2_O_5_ addition is referred to as 0%. The electrodes were connected to an electrode potential-amplifying system with an ultralow-input bias current operational amplifier AD549JH (Analog Devices, Wilmington, MA, USA). The amplifying system was connected to a computer through the data acquisition board AD302 (Data Translation, Inc., Malboro, MA, USA). Polymyxin B (PMB) was from Sigma-Aldrich, and phenyldicarbaundecaborane potassium salt was synthesized and purified by Dr. S. Sutkuvienė (Vytautas Magnus University).

The ATP content of the cells was determined by the luciferin–luciferase method using an ATP biomass kit (BioThema) [21]. The cells were incubated as described for the electrochemical measurements. A 25 μL sample of the suspension was withdrawn and mixed with 750 mL of 50 mM Tris-HCl, pH 7.0, and 200 μL of the ATP monitoring reagent. The light emission of the sample was measured with a Turner BioSystems 20/20 luminometer (Promega). The extractant (25 μL) and ATP reagent (100 μL) from the kit were added to the sample using two separate injectors. The light emission of the intracellular ATP content was determined after 60 s (to ensure that the cell envelope was destroyed). For calibration, 25 μL of ATP standardsolution (100 nM) was used. 

For the turbidity measurements, 80 μL samples were taken every 5–10 min throughout the experiments. A representative set of curves from three to four independent simultaneous measurements of the electrochemical parameters, ATP content, and turbidity of the taken samples is presented.

Data analysis. The numbers of CFUs and PFUs are presented as means ± standard deviation. The representative sets of curves from 3 to 5 independent series of measurements are presented in the figures.

## 3. Results

### 3.1. Infection Cycle

**Figure 1:**
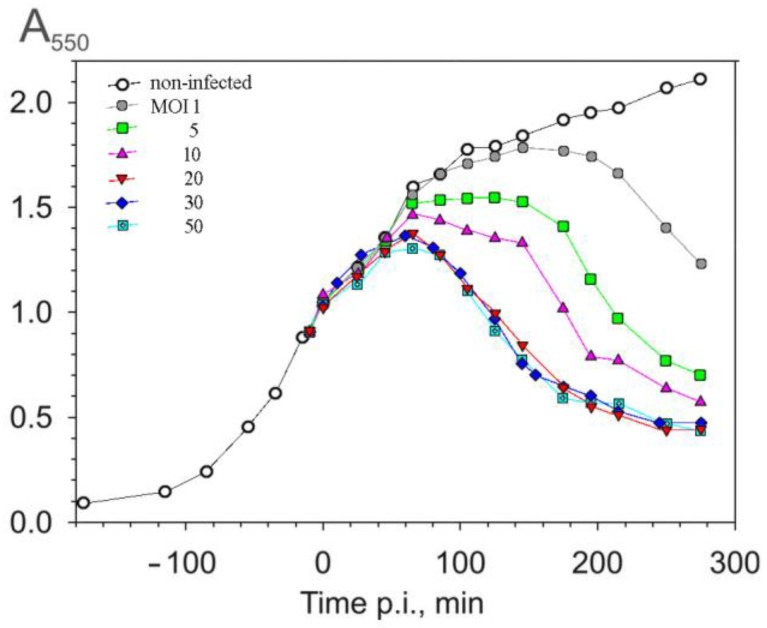
Dependence of the duration of a one-step infection cycle on the multiplicity of infection (MOI), monitored using culture turbidity measurements. *P. aeruginosa* cells were grown at 37 °C with aeration to 7 × 10^8^ CFU/mL (A_550_ = 1) and infected using the MOIs indicated in the figure.

One-step infection cycle experiments were carried out to evaluate the influence of the multiplicity of infection (MOI) on the duration of the infection cycle and on the amount of virus particles released. As shown in Figure 1, no significant difference in the infection dynamics was observed when an MOI of ≥ 20 was used. In all cases, lysis began ~70–75 min p.i.

At lower MOIs, a period of absorbance stabilization was observed before the drop in turbidity of the suspension, which may reflect non-synchronous infection. Two rounds of phage production could give rise to the duration of the infection cycle observed with MOIs of one or five. Infection is, probably, both non-synchronous and variable, with individual cells infected by 0–20 phage particles at an MOI of five. The lysate titers (~2 × 10^11^ PFU/mL) were similar among the tested MOIs, and approximately 300 infective particles were released per cell under these conditions. An MOI of 20 was chosen for further experiments.

### 3.2. Artificial Lysis of PRR1-Infected Cells

**Figure 2:**
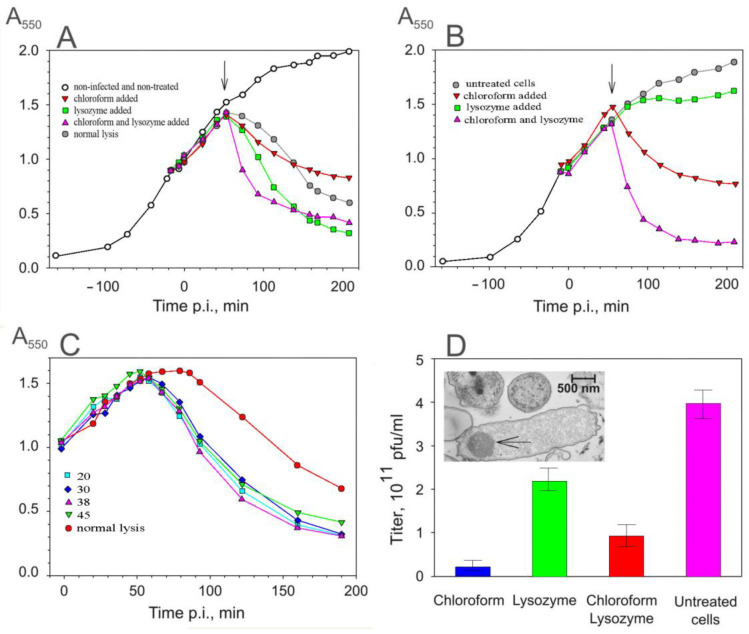
Artificial lysis of *P. aeruginosa* cells. (**A**) PRR1-infected cells; (**B**) uninfected cells. The arrows indicate the time points when chloroform, lysozyme, or both were added. (**C**) Sensitivity of the infected cells to lysozyme. Enzyme was added to the cell suspension at the figure-indicated time points. (**D**) Effect of chloroform, lysozyme, or both on the number of infective phage particles in the lysate from the experiment, as presented in (**A**). Insert represents thin-section EM of lysing cells (100 min p.i.) with PRR1 particles clustered inside (arrow).

ssRNA phage-induced cell lysis is slow compared to many other phage systems [14]. Additions of chloroform, lysozyme, or both were tested for their ability to enhance the lysis process. The results presented in Figure 2A show that lysis was accelerated when chloroform (damaging membranes) and/or lysozyme (hydrolyzing peptidoglycan) were added. However, as shown in Figure 2B, lysozyme alone was not able to lyse uninfected PAO1 cells and required the addition of chloroform. The chloroform-induced drop in the turbidity of the uninfected and infected cultures was fast, but the final level of turbidity stayed higher than after normal lysis. The most efficient drop in turbidity was achieved when lysozyme and chloroform were added simultaneously to the infected culture.

The sensitivity of the infected cells to lysozyme indicates that there are lesions in the OM that allow the lysozyme to access the peptidoglycan layer. To explore the kinetics of an infection-induced increase in OM permeability, the cell sensitivity to lysozyme was assayed at different stages of the infection cycle. As shown in Figure 2C, irrespective of when the lysozyme was added, its effect was observed 60 min p.i., approximately 15–20 min before the initiation of normal lysis.

The phage concentration in the culture supernatant after chloroform- and lysozyme- or, especially, chloroform-only-induced lysis (Figure 2D) was somewhat less effective than that obtained after normal or lysozyme-induced lysis. Since the virus is stable in the presence of chloroform, it seems that chloroform caused lesions in the cell envelope that compromised the release of PRR1. A possible cause of the limited release of PRR1 particles is seen in the thin-section EM image (Figure 2D, insert). The PRR1 progeny forms aggregates containing some 4000 particles (assuming that the aggregates are spherical), and the release of the aggregates is hindered by the cell envelope. Such intracellular virus aggregates have also been observed in cells infected with other members of the *Leviviridae* family [22]. 

### 3.3. The Accumulation of Infective Virus Particles

**Figure 3:**
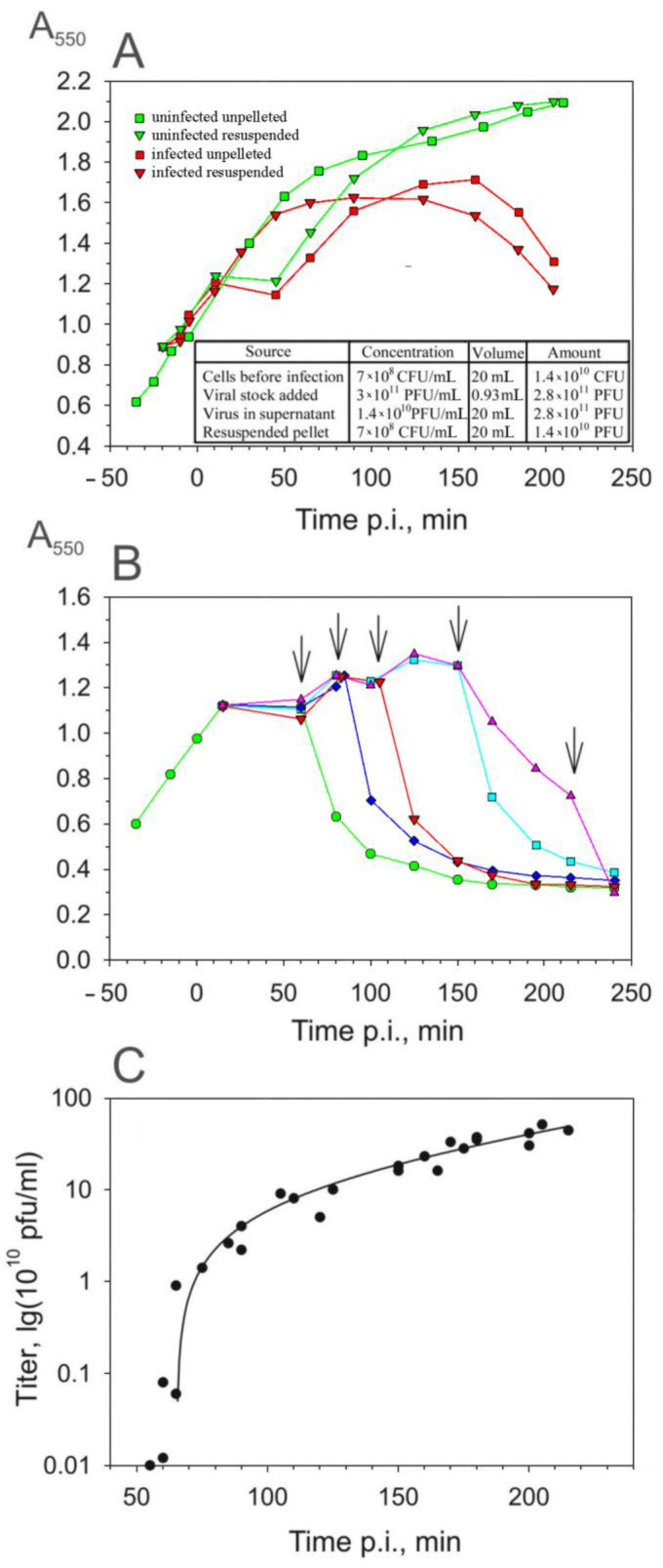
Production of infectious PRR1 particles during the one-step infection cycle. (**A**) Influence of the removal of unadsorbed phage particles (centrifugation and cell resuspension) on cell growth and the course of infection. (**B**) Lysis caused by chloroform and lysozyme added at time points p.i., as indicated by the arrows. (**C**) Infective PRR1 particles released by artificial lysis. The timing of the infection cycle (*x*-axis) includes the 35 min needed to remove the unadsorbed phage particles.

Although the aggregates made it difficult to assay the PRR1 yield, the kinetics of the viral appearance in the host cytosol could be assayed by titering the supernatants of the artificially lysed cells. To increase the sensitivity of this assay, unadsorbed (released pilus-associated and free) PRR1 particles were removed from the infection mixture by pelleting the cells (see Section 2). This procedure did not have any adverse effects on cell growth or virus production (Figure 3A), and practically all the unadsorbed virus particles were removed during centrifugation (Figure 3C). The results of the titering of the supernatant showed that the irreversible binding of PRR1 particles to the host surface is a slow and ineffective process (Figure 3A, insert).

The plating of the resuspended cell suspension on a PAO1 lawn indicated that at an MOI of 20, 15 min is sufficient to infect all the sensitive cells, e.g., the number of infective centers obtained was proportional to the number of cells present (Figure 3A, insert).

At different time points p.i., chloroform and lysozyme were added simultaneously to the suspension of resuspended cells to cause premature lysis (Figure 3B). The number of released PFUs increased approximately 100-fold between 55 and 60 min p.i., reflecting the accumulation of the mature virus particles inside the asynchronously infected cells (Figure 3C). Taking into account that the removal of unadsorbed phage particles, which took ~35 min (Figure 3A), it is possible to conclude that the first infective PRR1 particles appeared in the cytosol ~20 min p.i. The number of PFUs in the lysate continued to increase as the culture turbidity decreased, indicating that the lysis of the infected cells was not synchronous and/or that the initial drop in turbidity reflects swelling of the infected cells rather than lysis. The addition of chloroform and lysozyme at the very end of the lysis period increased the virus titer approximately two-fold. Under these conditions, an average of 600 infective phage particles were released from each infected cell (see also aggregates in Figure 2D). However, as described in the following sections, both the latent period and the burst size are strongly dependent on the growth conditions.

Lysis-related physiological changes in the host cells. The entire infection cycle was monitored online in thermostated and aerated reaction vessels using the same infection conditions as in the flasks [23]. Both virus production and changes in culture turbidity coincided with the one-step infection curves obtained in the flask cultures, indicating that the infection cycle was not altered when the reaction vessel was used.

**Figure 4:**
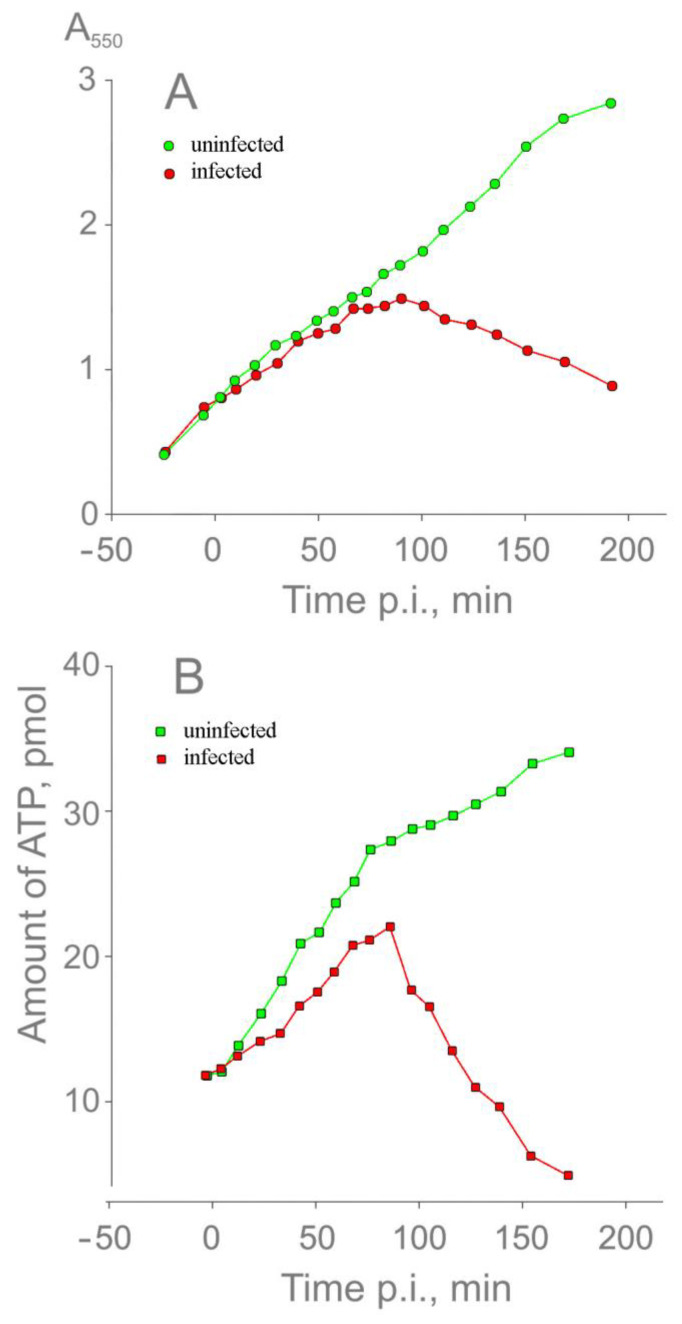
Changes in the suspension turbidity (**A**) and ATP content (**B**) of the cells during a one-step infection cycle. Cells were grown in a flask to a concentration of 5 × 10^8^ CFU/mL, transferred into a thermostated vessel (37 °C), incubated for 10 min, and infected with PRR1 at an MOI of 20. Also, 80 μL samples for A_550_ and 5 μL samples for ATP measurements were taken at different time points directly from the vessels.

To follow the energy state of infected cells, the ATP content of the cell suspension was measured during the infection cycle. During the first 60 min of infection, the turbidities of the infected and the uninfected suspensions were almost identical (Figure 4A). However, in infected cells, the amount of intracellular ATP increased considerably slower than in uninfected cells and started to decrease synchronously with the drop in turbidity (Figure 4B).

**Figure 5:**
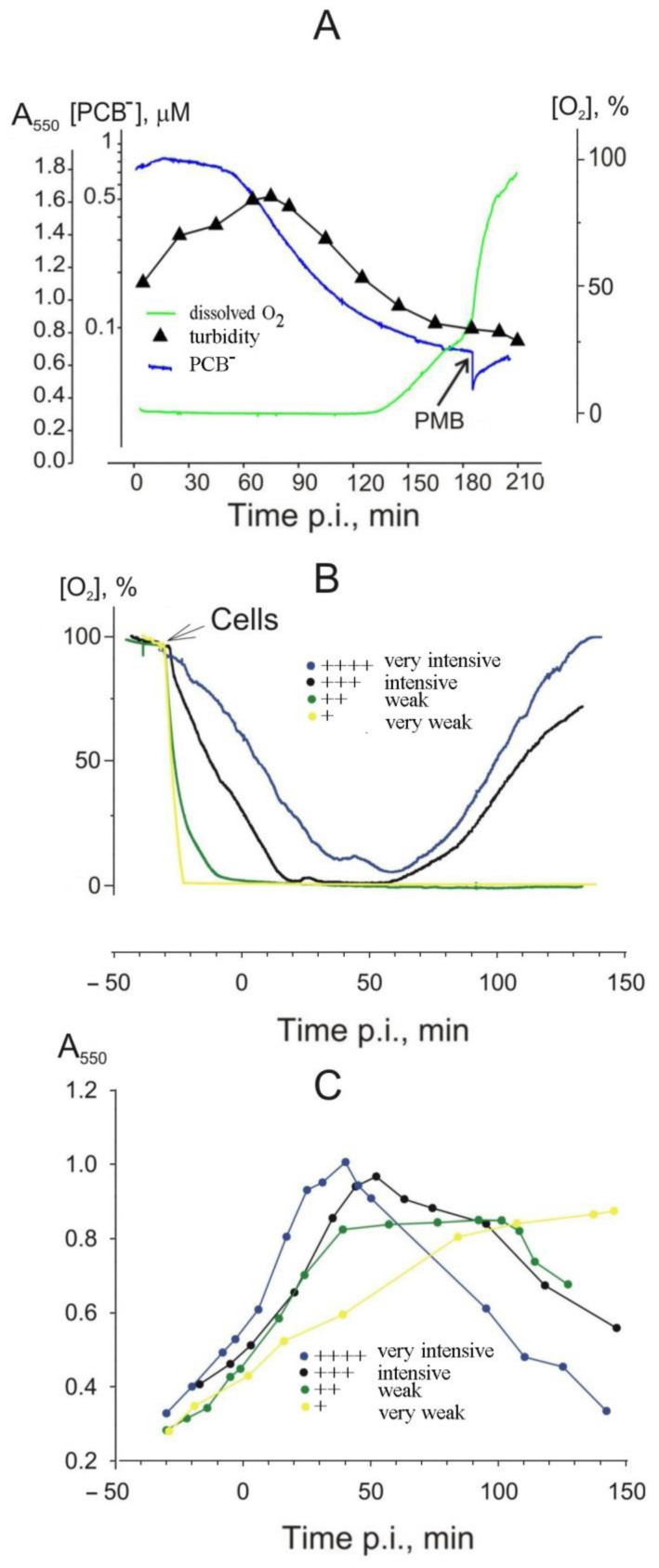
Influence of aeration on the course of the infection cycle. (**A**) Cells were grown in a flask to a concentration of 6 × 10^8^ CFU/mL and transferred into the thermostated vessel (37 °C), and PCB^-^ was added to a final concentration of 1 μM. The suspension was incubated for 10 min and infected with PRR1 at an MOI of 20. The medium concentrations of PCB^−^ and dissolved oxygen were monitored as described in Materials and Methods. For monitoring culture turbidity, 80 μL samples were taken from the vessel. At the end of the experiment, polymyxin B was added to a final concentration of 25 μg/mL. For dissolved oxygen (**B**) and turbidity (**C**) measurements, the cells were grown to 7 × 10^8^ CFU/mL, pelleted, and resuspended in a minimal volume of LB. The concentrated suspension was added to fresh LB (37 °C) in the vessel to obtain A_550_ ≈ 0.3, incubated for 10 min, and infected at an MOI of 20. Different levels of aeration are represented by different colored curves. The culture turbidity was monitored as in (**A**).

The metabolic activity of the bacteria and the state of the cell membranes during the infection cycle were measured by monitoring the dissolved oxygen and phenyldicarbaundecaborane (PCB^−^) concentrations in the medium (Figure 5A). PCB^−^ is a lipophilic anion that accumulates in the membranes of metabolically inactivated bacteria and is used to distinguish between damaged and intact cells [20,23]. Intact, uninfected cells bound PCB^-^ weakly, and the addition of PRR1 particles to the cell suspension did not increase the binding. However, perturbations in the cell envelope during the later stages of PRR1 infection damaged the envelope barrier to PCB^−^, allowing this lipophilic anion to bind to the cellular membranes. The decrease in the PCB^−^ concentration in the medium started at the same time that the infected cells became sensitive to lysozyme (see Figure 2C), approximately 20 min earlier than the decrease in turbidity (Figure 5A). Consequently, an increase in PCB^-^ binding is one of the earliest indications of the initiation of events leading to lysis. 

We also monitored the physiological state of the infected cells by monitoring oxygen consumption. However, at the initial cell density of 7 × 10^8^ CFU/mL, which was optimal for virus growth (see also [16]), the concentration of dissolved oxygen in the culture medium during most of the infection cycle was below the detection level of the electrode.

For this reason, no apparent response in the assimilation of oxygen was observed until the very end of lysis (Figure 5A). These experiments indicate that, even under intense aeration, because of the intense respiration, the cell suspension is not saturated with oxygen. As a result, the initiation of lysis does not result in an immediate increase in the concentration of oxygen in the medium, as all the dissolved oxygen is consumed by the remaining cells.

To monitor the consumption of oxygen more accurately, the initial cell density was reduced to 2 × 10^8^ CFU/mL (A_550_ ≈ 0.3), and the level of aeration was varied by changing the rate of magnetic stirring. As shown in Figure 5B, the slowest stirring resulted in the rapid depletion of the dissolved oxygen from the medium, and no changes in the dissolved oxygen concentration were observed during the infection cycle. On the contrary, intensive aeration resulted in a slower decrease in the concentration of dissolved oxygen during the infection cycle, and it was at the measurable level during the entire infection period. No alterations in cellular respiration were induced by PRR1 at the early stages of the infection. A decrease in oxygen consumption was observed when the cells entered the lytic stage. Interestingly, an increase in the dissolved oxygen concentration started a few minutes after the decrease in culture turbidity (Figure 5B). 

Altering the intensity of aeration allowed us to observe differences in the length of the infection cycle and the kinetics of the lysis (Figure 5B,C). The lysis of the well-aerated cultures started earlier and at a higher cell density than the poorly aerated cultures. The well-aerated suspensions of the infected cells started to lyse at 40–55 min p.i. In contrast, the lysis of the poorly aerated cultures was significantly delayed (100 min p.i.) or not observed at all, although a considerable amount of the reproduced phage particles was detected after artificial lysis using lysozyme and chloroform. Even so, the poorly aerated cells treated with chloroform and lysozyme released up to ~400 infective phage particles per cell, whereas the well-aerated cells were able to release up to ~1000 infective phage particles per cell via natural lysis. At a high level of aeration, the duration of the latent period was shortened, but it did not change the rate of normal lysis (Figure 5C).

### 3.4. Effects of Salts and Metabolic Poisons on PRR1 Infection

**Figure 6:**
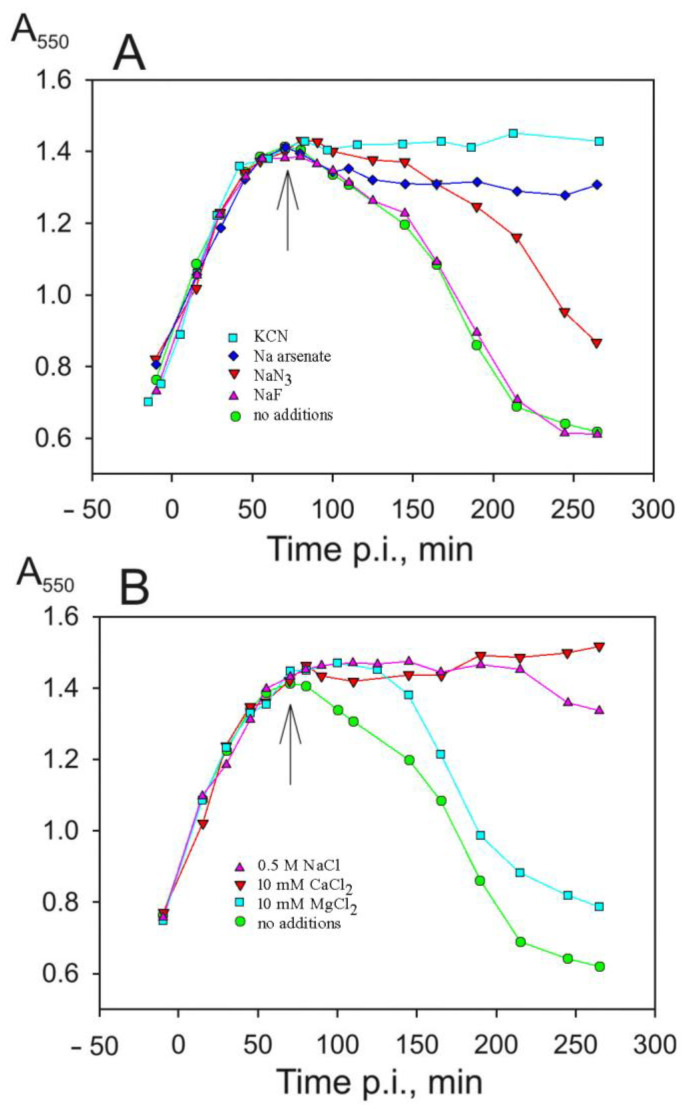
Effects of metabolic inhibitors and other salts on the turbidity of infected *P. aeruginosa* cultures. All medium supplements were added (as indicated by an arrow) 10 min before the predicted start of lysis. Concentrations of metabolic inhibitors (**A**) were 20 mM, and concentrations of the added salts (**B**) are indicated in the figure.

Metabolic poisons, including KCN, sodium arsenate, NaN_3_, and NaF, were tested at 20 mM concentrations (Figure 6A). NaF, an inhibitor of glycolysis, had no apparent effect on the lysis of the infected cells. The addition of NaN_3_, known to block respiration and ATP hydrolysis by membrane H^+^-ATPase, resulted in halted lysis. KCN, which blocks respiration by inhibiting terminal oxidase, and arsenate, which reduces the intracellular ATP pool via an arsenolysis reaction, completely inhibited lysis.

Interestingly, the course of infection was not considerably affected by an increased Mg^2+^ concentration in the medium to 10 mM (Figure 6B), which is known to inhibit *E. coli* cell autolysis [24]. Instead, the lysis was effectively inhibited by the addition of 10 mM of CaCl_2_. Increasing the ionic strength and the osmotic pressure of the medium by adding NaCl (final concentration 0.5 M) effectively prevented cell lysis (Figure 6B) and the release of the virus.

### 3.5. PRR1 Lysis Gene Expressed in E. coli

The lysis gene was predicted from the PRR1 genome sequence, and its activity was confirmed by the expression of the protein in *E. coli* [15]. Here, the cDNA of the lysis gene (nt region 1780–1944) was generated and inserted into plasmid pJJ2 [25]. The resultant plasmid was designated pTR117 and transferred into *E. coli* HMS174(DE3). The induction of gene expression was achieved using 1 mM IPTG Lysis of HMS174(DE3)(TR117) but not of HMS174(DE3)(pJJ2) indicated that the product of this gene is necessary and sufficient for lysis.

**Figure 7:**
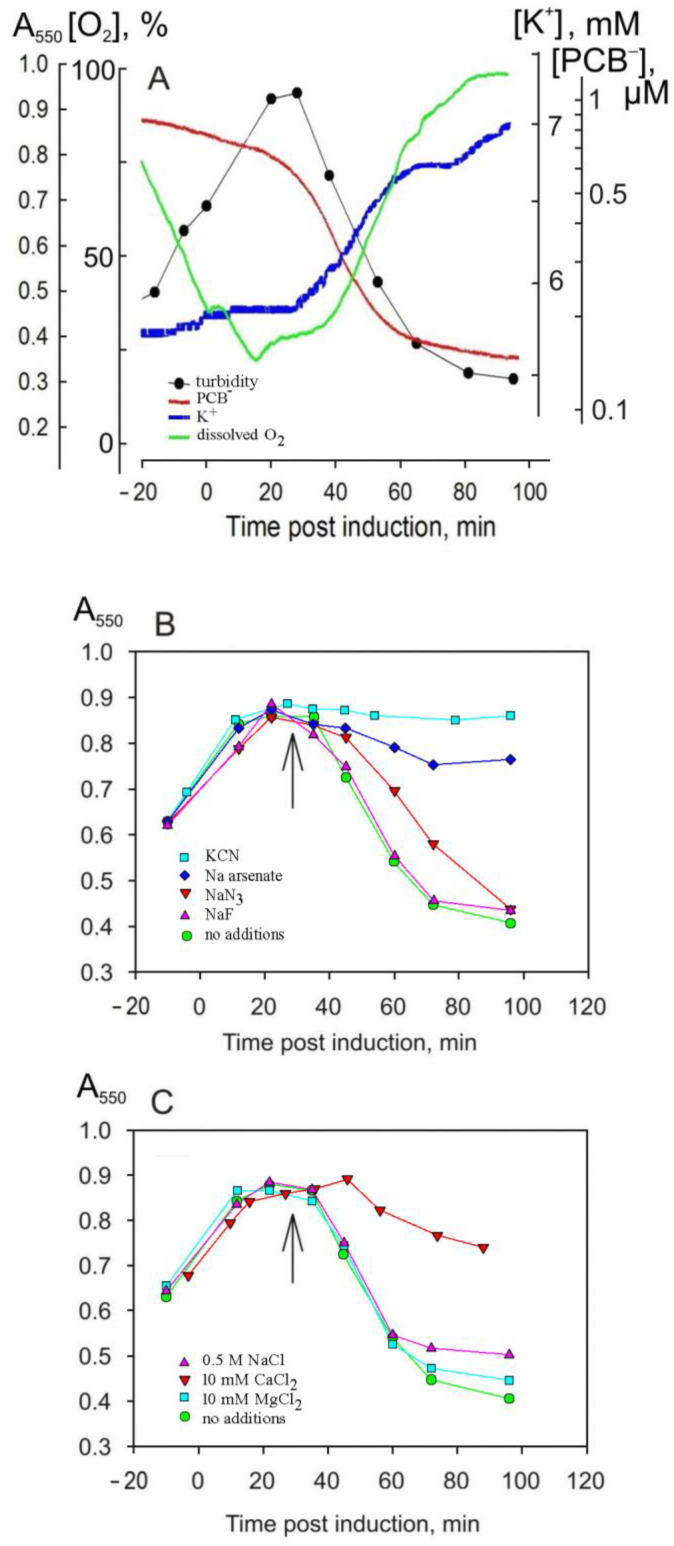
The lysis of *E. coli* HMS174(DE3) cells carrying a recombinant plasmid with the PRR1 lysis gene. Changes in culture turbidity and medium concentrations of dissolved oxygen, PCB^−^, and K^+^ (**A**); effects of 20 mM of metabolic poisons (**B**) and effects of different salts at indicated concentrations (**C**). Gene expression was induced by adding 1 mM of IPTG; additions of poisons and other salts to the cell suspension are indicated by arrows. Concentrations of metabolic inhibitors (**B**) were 20 mM, and concentrations of the added salts (**C**) are indicated in the figure.

For the characterization of the induced lysis in *E. coli*, the same physiological parameters were measured as in the PRR1 infection of *P. aeruginosa* cells (Figure 7A). In addition, the lysis protein-induced leakage of intracellular K^+^ ions was monitored. The decrease in the turbidity of the *E. coli* cell suspension and the leakage of K^+^ started ~25–30 min post-induction. PCB^−^ binding by the cells started ~5 min earlier (20 min post-induction) than the decrease in the culture turbidity, while the increase in the dissolved oxygen concentration in the medium (the weakening of cellular respiration) started later (35 min post-induction; Figure 7A). As in the case of the PRR1 infection-mediated lysis of *P. aeruginosa* cells, the dependence of lysis protein-induced events on aeration was observed: reduced stirring or increased cell density resulted in a prolonged infection cycle. 

As shown in Figure 7B, the effects of metabolic poisons on the course of lysis were the same as in the PRR1 infection: NaF had no apparent effect; NaN_3_ resulted in prolonged lysis; and KCN and sodium arsenate efficiently inhibited lysis. However, the effect of Ca^2+^ on the lysis protein-induced destruction of *E. coli* cells was less pronounced, and the inhibitory effect of the increased NaCl concentration was negligible in the *E. coli* expression system compared to the PRR-1 infection of *P. aeruginosa* cells. The inhibitory effect of Mg^2+^ ions on cell autolysis and lysis gene-mediated cell lysis is usually considered to be associated with the stabilization of spheroplasts [26]. However, 10 mM of Mg^2+^ had no inhibitory effect on PRR1 lysis protein-induced *E. coli* cell disruption (Figure 7C).

## 4. Discussion

The bacteriolytic E protein of the ssDNA phage ϕX174 is responsible for host cell lysis, and its molecular target has been well examined. Protein E inhibits an integral membrane protein involved in peptidoglycan synthesis, phospho–MurNAc–pentapeptide translocase [10,27]. Similarly, in the ssRNA phage MS2, a close relative of PRR1, virus release is promoted by the induction of the host autolytic system, which is achieved by a specific insertion of the L (lysis) protein into the cell envelope [7]. Protein L- and protein E-mediated localized lysis results in empty cell envelopes (ghosts), which retain most of the host cell’s morphology [7,28]. In contrast, phages that use the holin–endolysin–spanin lysis system yield completely disrupted cells. However, MS2- and ϕX174-induced lysis differ in some respects. E protein-mediated lysis, which is characterized by fused plasma and outer membranes, releases more than 90% of the cytosolic enzymes but only about 12% of the periplasmic ones. In contrast, L-mediated lysis liberates the same amount of cytosolic markers but as much as 80% of the periplasmic L-lactamase [29]. The turbidity of the PRR1-releasing cell suspension stays considerably higher compared to, i.e., the dsDNA phage PM2 reproducing in Gram-negative *Pseudoalteromonas* sp. cells after natural, chloroform-, or inhibitors of energetical metabolism-induced premature lysis [21].

We observed here that PRR1 infection or the expression of the lysis protein from the plasmid renders PAO1 cells sensitive to lysozyme approximately 10–20 min before the start of normal lysis. At the same time, PCB^-^ concentration in the medium starts to decrease, also indicating damage to the OM barrier. If this time point corresponds to the insertion of a synthesized lysis protein into the cell envelope, such an insertion does not permeabilize the energetically active PM. K^+^ leakage, indicating an increase in the permeability of the PM of the lysis protein-expressing cells, starts ~10 min later than the permeabilization of the OM. Low concentrations of the lysis protein in the cell envelope could cause stress and the dissociation of bonds between the OM and peptidoglycan layer, leading to lesion formation in the OM [12] and vesicle release [30]. It is possible that a high amount of lysis protein is necessary to form the permeabilizing structures in the PM, like holin protein does at the end of infection by dsDNA-containing phages [4]. The aTP content of the infected cells starts to decrease simultaneously with the turbidity of the bacterial suspension, indicating that an increase in the OM permeability does not affect the permeability or PM-dependent ATP synthesis.

It is known [31] that cell growth is required for phage MS2 L-protein-mediated cell lysis and virus release. The results of our experiments indicate that infected *P. aeruginosa* cells start to lyse earlier when they grow under conditions of intense aeration. Under these conditions, when the duration of the latent period is shortened, there is a considerable increase in the yield of the reproduced phage. Improved aeration also enhances the growth rate of the uninfected cells. Our results support the observations of Alvarez-Ortega and Harwood [32] that *P. aeruginosa* cells can grow effectively at low dissolved oxygen concentrations. The experiments with *P. aeruginosa* cells confirm that the detection of and reduction in the dissolved O_2_ with a Clark-type oxygen electrode is less effective than the consumption of O_2_ by various aerobic bacteria [21,23]. However, medium-dissolved oxygen concentrations sufficient to support *P. aeruginosa* cell growth do not assure efficient lysis. The poorly aerated infected cultures lysed considerably later (>100 min p.i.) or did not lyse at all during the period of monitoring, although the results of the artificial lysis showed that the synthesis and assembly of the infective phage progeny were only 2–3 times less effective. The experiments with the phage ϕX174 indicated that the lysis was dependent on the presence of a proton motive force 3 to 5 min before lysis [33]. The results of our experiments at various intensities of aeration indicated that, despite the earlier beginning of lysis, the dynamics of the decrease in suspension turbidity do not depend on the level of aeration.

*P. aeruginosa* is usually described as a bacterium that favors aerobic growth conditions [34]. Still, they can survive anaerobically for several weeks by pyruvate fermentation in the absence of nitrate, nitrite, or arginine [35]. In the presence of nitrate, anaerobic respiration abolishes pyruvate fermentation [36]. However, pyruvate fermentation allows only the survival and not the growth of P. aeruginosa cells during anaerobic energy starvation conditions [36]. In our experiments at the conditions of the lowest aeration, the uninfected P. aeruginosa cultures grew, reproducing PRR1 after infection, but the release of the phage particles was halted.

The MS2-infected cells captured in the transmission and scanning electron micrographs showed various morphological aspects of the intermediates in the lysing process [12,37]. In contrast to endolysin–holin–spanin-mediated lysis, single-lysis protein-induced cell envelope degradation is limited and observed only locally [6,7,31]. The release of PRR1 virions is further obstructed because PRR1 particles tend to form large, ordered aggregates inside the cell, as do other members of *Leviviridae*. The combination of chloroform and lysozyme results in the most efficient premature lysis of infected cultures, and such treatment late in the infection cycle yields a two-fold increase in the released PFUs. However, such artificial lysis resulted in only a 50% yield of phage particles compared with lysis induced with lysozyme alone, but chloroform-induced lysis resulted in an even lower number of progeny phages. Consequently, it appears that chloroform-induced perturbations in the cell envelope disturb PRR1 release despite enhancing cell lysis. Thus, it is likely that an intact PM is an important prerequisite for the efficient release of phage progeny. Lysing agents destroy cells more effectively but less selectively, and some infected cells are lysed prematurely before the maximal number of particles have assembled. Natural lysis takes more time because only ready-for-lysis cells are destroyed; in such cases, more virus particles are assembled per cell.

As the accumulation of phage PRR1-encoded lysis proteins but not the assembly of viral particles induces cell lysis, it is unclear how the timing of lysis is controlled. In the case of three-component lysis systems, the addition of metabolic poisons late in the infection immediately triggers premature lysis, indicating that the precise regulation of endolysin–holin–spanin-mediated cell wall disruption depends on the energy state of the PM [4,5]. In contrast, during PRR1 infection, the blockage of respiration by KCN or a reduction in the intracellular ATP concentration by arsenate inhibited the lysis. The same effect was observed when the synthesis of the PRR1 lysis proteins was induced in an *E. coli* expression system. Such experiments would not be possible with bacteriophages infecting *Bacillus subtilis* or other Gram-positive bacteria, as the inhibition of respiration or an increased concentration of salts induces the autolysis of these cells [38]. The prevention of the lysis of infected cells after the supplementation of the infection medium with a high concentration of NaCl could be a result of the dysregulation of the assembly of phage particles or the inhibition of genome packaging. Most probably, not only the dsDNA virus genome packaging [39] but also the placement of ssRNA into the PRR1 capsid is sensitive to the osmotic pressure of the infection medium, as the addition of 0.5 M of NaCl to the medium should increase the intracellular K^+^ and Na^+^ concentrations [40]. However, this does not prevent PRR1 lysis gene-expressing *E. coli* cell lysis.

The results of our experiments suggest that the PRR1 lysis protein plays the role of spanin, the OM permeabilizing protein, in dsDNA phage-induced lysis. An increase in OM permeability is the earliest event observed in PRR1 infection, as well as in the case of the phage MS2 [14]. The peptidoglycan of the infected cells could be digested by the cellular autolysins after the PRR1 lysis protein-induced loss of control of these enzymes in the infected cells. However, *E. coli* has ~40 different proteins that synthesize and hydrolyze peptidoglycan in the periplasm, and our understanding of their role is still incomplete [41].

Our results, depicting a reduced latent period at lower cell densities, are not consistent with theoretical models that predict longer latent periods at low cell densities [42,43,44]. These optimality models predict that a trade-off exists between the latent period and burst size. The elongation of the latent period should ensure the accumulation of newly produced phage particles. However, the results of the PRR1-induced lysis are in good agreement with the results obtained in studies of the holin-dependent lysis systems of phages PM2 [21] and Bam35 [23]. In these virus systems, higher burst sizes are obtained at lower densities of infected cells, suggesting that enhanced nutrient and oxygen levels improve the energy state and enhance the metabolic activity of the host, resulting in a more efficient production of virus particles.

## 5. Conclusions

The on-line monitoring of the entire infection cycle showed that the single-gene-mediated lysis of phage PRR1-infected cells strongly depends on the physiological state of the host cells. PRR1 infection renders *P. aeruginosa* cells sensitive to lysozyme approximately 20 min before the drop in suspension turbidity starts. At the same time, the infected cells started to accumulate lipophilic anions. However, such an increase in the outer membrane permeability did not affect the functions of the plasma membrane. The same effects were observed when the synthesis of the PRR1 lysis protein was induced in an E. coli expression system. The blockage of respiration or a reduction in the intracellular ATP concentration during the infection resulted in the inhibition of lysis. Dissolved oxygen concentrations sufficient to support cell growth did not ensure efficient lysis, and a coupling between the initiation of cell lysis and the aeration level of infected cells was observed. However, the duration of the drop in suspension turbidity did not depend on the level of aeration.

## Data Availability

The data presented in this study are available on request from the corresponding author.
